# Photocurable Hypervalent Fluorinated Sulfur Containing Thin Films with Remarkable Hardness and Modulus

**DOI:** 10.3390/molecules29184413

**Published:** 2024-09-17

**Authors:** Kelly A. Bonetti, Deniz Rende, Michael Murphy, John T. Welch

**Affiliations:** 1 Department of Chemistry, University at Albany SUNY, Albany, NY 12222, USA; kbonetti@albany.edu; 2 Department of Materials Science & Engineering, Rensselaer Polytechnic Institute, Troy, NY 12180, USA; rended3@rpi.edu; 3 Department of Nanoscale Science & Engineering, University at Albany SUNY, Albany, NY 12222, USA; michael.murphy@us.tel.com

**Keywords:** photochemistry, polymerization, fluorine, sulfur, modulus, hardness

## Abstract

Novel tetrafluoro-λ^6^-sulfanyl-containing oligomers prepared by visible light-promoted addition of 1,4-(bis-chlorotetrafluoro-λ^6^-sulfanyl) benzene or 1,3-(bis-chlorotetrafluoro-λ^6^-sulfanyl) benzene to either 1,4-diethynyl benzene or the 1,3-diethynyl isomers form hard, stress resistant thin films on spin casting. The isomeric oligomers were utilized to establish a structure-function relationship for the mechanical properties of films prepared from the oligomers. The Young’s moduli of 145-nm-thick cured films could reach 60 GPa. The measured hardnesses, between 1.57 and 2.77 GPa, were more than double those of polymethyl methacrylate (PMMA) films. Curing of the tetrafluoro-λ^6^-sulfanyl-containing polymer films by UV irradiation resulted in coatings that exhibited remarkable hardness and modulus with good surface adhesion to silicon.

## 1. Introduction

It has been previously demonstrated that aryl tetrafluoro-λ^6^-sulfanyl chlorides (ArSF_4_Cl) [1], are photoreactive. Compact fluorescent light irradiation of ArSF_4_Cl in the presence of alkenes or alkynes readily leads to addition of ArSF_4_Cl across the multiple bond [2,3]. As neither ArSF_4_Cl or aryl alkynes appreciably absorb light above ca 300 nm, complex formation by the reactants was postulated to result insignificant absorption in the shorter wavelength region of light emitted by a compact fluorescent bulb [4]. The visible light-promoted addition of 1,4-(bis-chlorotetrafluoro-λ^6^-sulfanyl) benzene to 1,4-diethynyl benzene yielded an oligomer with an average molecular weight of 3–5 kDa, consisting of typically 8–10 repeat units, with a moderate dispersity (PDI) between 1.6–2.4 [4]. Exogenous photo-initiators were not required.

The mechanical properties of thin polymer films are crucial to the utility of these materials in thin film devices, in active and passive coatings and in flexible displays [5,6]. Surface adhesion, wettability, and thermal and mechanical resistance of the films are crucial products of the film chemistry [7]. Polymer thin films have found applications as dielectric insulators in semiconductor devices, in alignment layers of flat panel displays and as hole transport layers in photovoltaic devices [8]. Thin polymer films must also be durable for applications such as touch screen devices or non-stick, wear-resistant coatings for displays or as protective layers for functional coatings in photographic and magnetic devices [9]. The use of acrylate, epoxy, polyester, polyamide and thiol/ene photocurable systems is well-known [10,11,12,13,14,15,16,17,18,19,20,21].

New functional materials, such as those described herein, incorporating the tetrafluorosulfanyl group in an oligomer backbone may lead to films with superior properties. Integration of this sterically demanding and strongly electron withdrawing functional group may influence π–π stacking and unique surface interactions. Novel -SF_4_- containing oligomers constitute a new molecular scaffold with interesting mechanical properties.

### 1.1. Polymer Film Mechanical Properties

Thin polymer films with extreme hardness and a high modulus are useful as adherent protective coatings, as scratch resistant surfaces or in composite layers. Young’s modulus and hardness illustrate material response to external stress by reversible elastic and irreversible plastic deformation.

Young’s modulus (E), a measure of the resistance of a material to elastic deformation when subjected to tensile or compressive stress, describes the extent to which the material will deform under a given stress within the elastic range. In mathematical terms, Young’s modulus is the ratio of stress to strain in the elastic deformation region. A film with a high Young’s modulus indicates the film is not easily distorted [22].

Hardness quantifies the resistance of a solid material to permanent deformation. Indentation hardness is approximately three times the yield strength [22], the mechanical property of a material that represents the level of stress at which the material begins to deform plastically. In other words, the yield strength is the point at which the material transitions from elastic to plastic deformation.

#### 1.1.1. Modulus

The flexibility of the polymer backbone, polymer molecular weight, the degree of crystallinity, and the extent of cross-linking all influence the modulus [23]. When measured by nanoindentation (NI), the stiffness of thin polymer films or coatings is typically less than 10 GPa and is dependent on the solid support. For example, the elastic moduli of thin films of poly(methyl methacrylate (PMMA), polystyrene (PS), polycarbonate (PC) and poly(vinyl chloride (PVC) on a silicon substrate have been reported to be 5.9 GPa, 5.2 GPa, 3.0 GPa, and 3.9 GPa, respectively [5]. Accurate measurement of the elastic properties of polymer films spun cast on hard materials like silicon can be difficult as the measured moduli are often higher than those found when bulk materials are examined [24]. In this work, the properties of films of the new oligomers determined by nanoindentation will be compared with those of PMMA films on the same substrate.

#### 1.1.2. Hardness

Bulk polymers typically have hardness values lower than 1 GPa but the hardness of thin polymer films coated on a hard substrate determined by nanoindentation can be as high as 3–10 GPa. For example, plasma deposited organosilicones, are reported to have hardnesses of 2.69 GPa [25]. The challenge of determining the mechanical properties of thin polymer films supported by a hard substrate is a consequence of the complex interactions of the plastic zone of the film with the substrate [23,26].

### 1.2. UV Curing of Thin Polymer Films

Photochemical curing of thin films enables the chemical alteration of the polymer without changing the morphology of the bulk material [27] and is commonly utilized in high performance coatings, adhesives, inks, and photoresists [15,28,29,30,31,32,33,34]. Photo-responsive thin films necessarily must bear reactive chromophores. UV irradiation of acrylates, epoxies, polyesters, thiol/ene systems and polyamides [10,11,12,13,14,15,16] is known to cross link reactive end groups. Irradiated polymer films thus are often stronger and more resistant to applied mechanical loads than uncured films. 

### 1.3. Summary

A novel oligomerization reaction of 1,4-(bis-chlorotetrafluoro-λ^6^-sulfanyl) benzene with 1,4-diethynyl benzene provides a new, flexible platform for photo-oligomerization reactions. Visible light promoted reactions obviate the need for higher energy sources of radiation and eliminate the need for exogenous photocatalysts. Catalyst free systems broaden the potential applications of the oligomeric products to include those applications where the presence of the catalyst in the product would be undesirable. Additionally, oligomer films may be cured by exposure to 200–450 nm light, yielding remarkably stiff and hard coatings. 

## 2. Results

### 2.1. Tetrafluoro-λ^6^-Sulfanyl-Containing Oligomers

**Figure 1:**
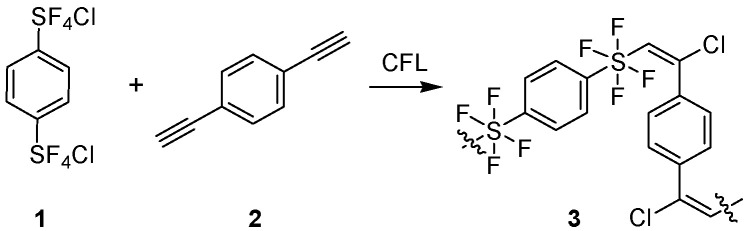
Illumination by a compact fluorescent bulb initiates the photo-oligomerization of a 1:1 ratio of two reactive co-monomers.

The visible light-promoted step growth polymerizations of bis-(chlorotetrafluoro-λ^6^-sulfanyl) benzene isomers with diethynyl benzene isomers yield radiation-sensitive oligomers [4]. Irradiation of a neat mixture of the co-monomers, e.g., **1** and **2**, or a solution of those co-monomers, with a compact fluorescent light source at room temperature, rapidly and efficiently formed oligomer **3** (Figure 1) without the need for exogenous photo-initiators. 

UV-triggered fast and non-toxic oligomerization [35] yields new functional films incorporating the strongly electron withdrawing and sterically demanding tetrafluorosulfanyl group. The backbone incorporation of this group can promote π–π stacking, resulting in a molecular scaffold that yields strong films with remarkable stress resistance.

### 2.2. Kinetics of Oligomerization

The photo-oligomerization of the co-monomers likely occurs by step growth addition, where the suitably functionalized termini of the co-monomers and the growing oligomers react. Both the co-monomers and the oligomers are present in the reaction mixture throughout the elongation process. Step-growth oligomers often grow by carbon–heteroatom bond formation whereas chain elongation in chain-growth polymerizations is typically driven by carbon–carbon bond formation. Samples of the oligomerization system, taken at various time points from an initial one-to-one mixture of the comonomers were analyzed by gel permeation chromatography (GPC).

**Figure 2:**
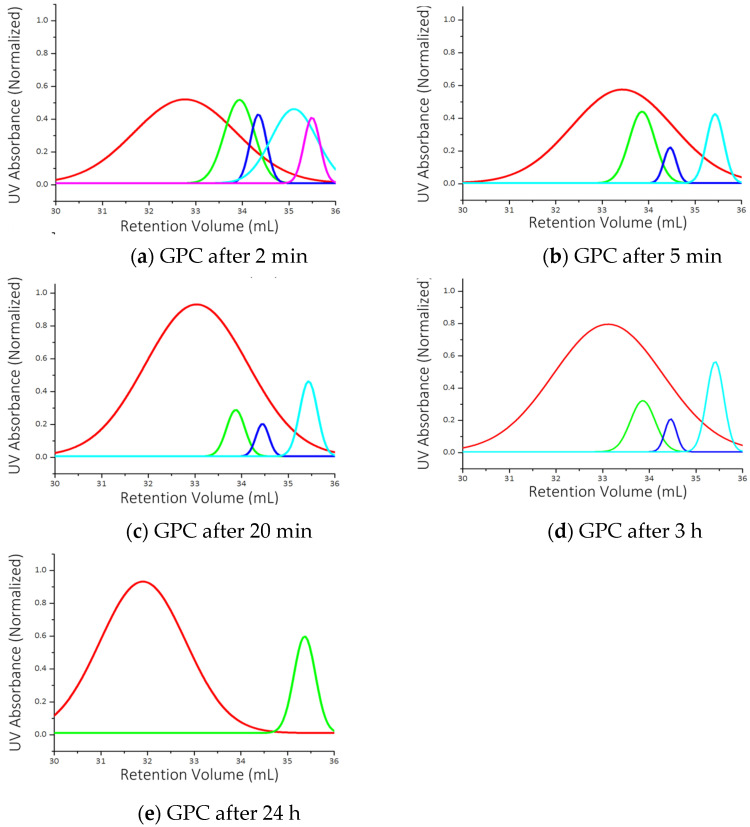
Kinetic study increasing the molecular weight of the formed reaction products. UV absorbance was measured at λ = 254 nm over five time points (**a**–**e**). The red trace can be attributed to the oligomer with the highest molecular weight. The other colored traces with the exception of the trace appearing with the longest retention requiring approximately 35.5 mL of eluant are derived from the formation of intermediate oligomers with increasing molecular weight. The longest retention trace is associated with the presence of unreacted diacetylene **2**. At increasing longer time points, the apparent concentration of lower molecular weight oligomers diminishes while some monomer **2** (35.5 mL retention) remains.

Low molecular weight oligomers were formed within 2 min (See Figure 2). Multiple low molecular weight components were present, including unreacted diacetylene up to 3 h after initiation. As the reaction proceeded, the quantities of lower molecular weight oligomers decreased until a higher molecular weight material was predominant. After 24 h, unreacted monomer remained, suggesting the oligomerization process terminated before complete consumption of the monomers. Longer reaction times and constant illumination were required for formation of the highest molecular weight product. 

Both reactive functional groups are required to interact for chain elongation, whether by reaction of a co-monomer with an oligomer or by reaction of the oligomers with one and another. Additionally, the photoaddition process of the aryl chlorotetrafluorosulfanyl group requires favorable orientation with the aryl alkyne for the π–π stacking interactions proposed to be essential for visible light absorption and subsequent photochemical addition of reactive end groups.

The disappearance of the dialkynylarene during the polymerization process was initially monitored by ^1^H NMR spectroscopy. It was found that the majority of the dialkyne was depleted within the first four hours of reaction, with the consumption of the dialkyne occurring most rapidly during the first hour. The co-monomers reacted quickly but joining of reactive end groups of the growing oligomers required increasingly longer reaction times, as would be predicted for a step growth mechanism.

**Figure 3:**
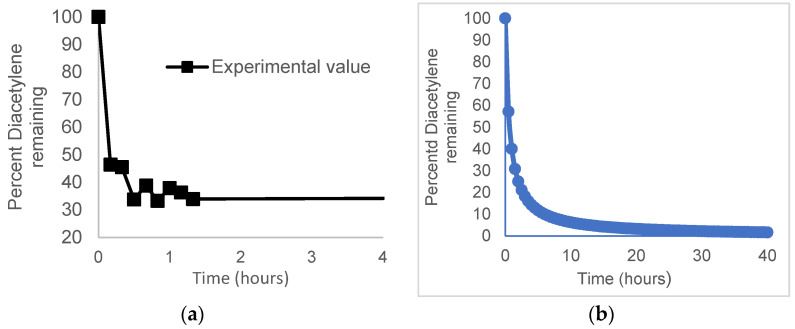
(**a**). The disappearance of dialkynylbenzene over time as determined by gas chromatographic analysis. The failure of the experimental values to reach zero reflect the termination of the oligomerization reaction by impurities in the chlorotetrafluorosulfanyl arene monomer. (**b**). The computed plot of the disappearance of the dialkynylbenzene as predicted by Equation (1).

As step growth polymerizations deplete monomeric units quickly, determination of the monomer concentration over the first hour of reaction was essential to establish the reaction order. It was most expedient to follow the depletion of the dialkyne monomer at frequent early time points by gas chromatography (See Figure 3). The results of these measurements were consistent with the trend observed by NMR described earlier. The fit of the experimental data is consistent with the addition reaction being most facile when both co-monomers were present and interacting effectively to form the intermediate charge transfer complex required for the absorption of visible light. These results are also consistent with the GPC kinetic experiments that indicated monomeric units are consumed rapidly early in the course of polymerization and only form longer oligomers at extended reaction times. The reaction was modeled using the following equation from manipulation of the integrated second order rate equation and the Carothers equation:

(1) $$\frac{M}{M_{0}}=\frac{1}{kM_{0}t+1}$$

where *M*_0_ = initial monomer concentration

*M* = monomer concentration remaining at time *t*

*k* = rate of the oligomerization

*t* = time of oligomerization

The percentage of diacetylene remaining can be computed by the fraction of monomer remaining.

### 2.3. Structure Function Relationship

**Figure 4:**
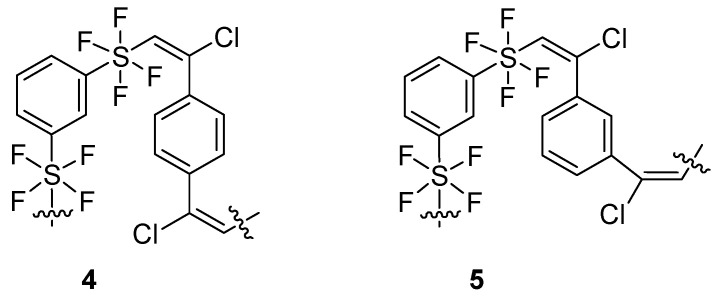
Isomeric tetrafluorosulfanyl-containing oligomers.

The direct addition of chlorotetrafluorosulfanyl arenes [1] to alkynyl arenes formed alkenyl aryl tetrafluorosulfanes [2] where steric and electronic effects help establish long range order [3]. Isomeric hypervalent fluorinated sulfur-containing oligomers **3**–**5** (Figure 1 and Figure 4) were prepared to evaluate the influence of isomer variations on the mechanical properties of photopolymer thin films.

While the isomeric oligomers have many similar properties, other properties such as dispersity, photoreactivity, and hardness are sensitive to the isomeric form.

### 2.4. Gel Permeation Chromatography (GPC)

Stepgrowth oligomers **3**, **4**, and **5**, consisting of approximately 8–10 repeats, have an average molecular weight between 3–6 kDa (See Figure S17–S19). The dispersities (Ð) between 1.6–2.4 were indicative of variable oligomer lengths (See Table 1).

### 2.5. Thermal Analysis

The thermal stability of the oligomers as well as the exothermic and/or endothermic thermal transitions that reflect changes in crystallinity or partial degradation were determined. The bulk oligomers **3**, **4**, and **5** exhibit two major weight losses on thermogravimetric analysis (TGA) as illustrated in Figure S1. The initial onset of decomposition occurs at 187 °C and is proposed to be a consequence of dehydrochlorination. Each of the oligomers demonstrate weight loss at nearly the same temperature suggesting similar chemistry occurs. Loss of HCl from each repeat unit corresponds to the modest 20% weight loss observed. The tetrafluorosulfanyl-containing oligomers undergo limited weight loss as temperatures increase, until approximately 500 °C, when the rate of weight loss increases dramatically.

#### 2.5.1. Thermal Gravimetric Analysis-Mass Spectrometry

**Figure 5:**
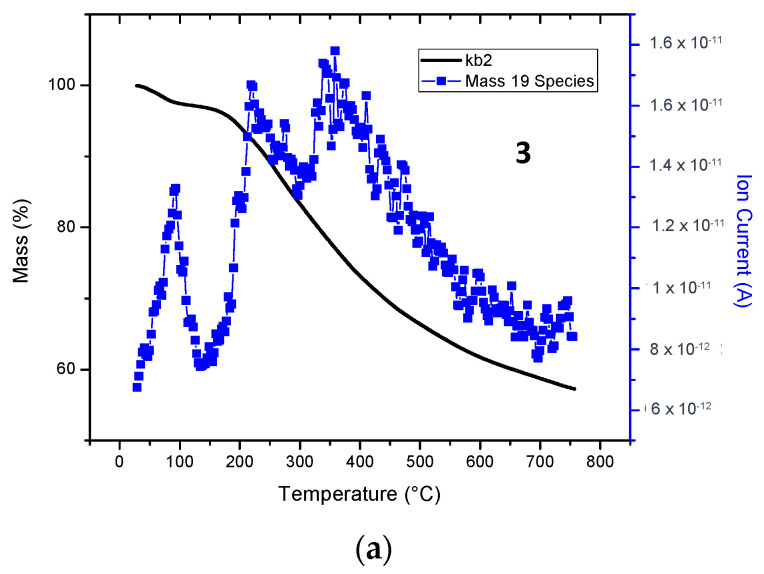

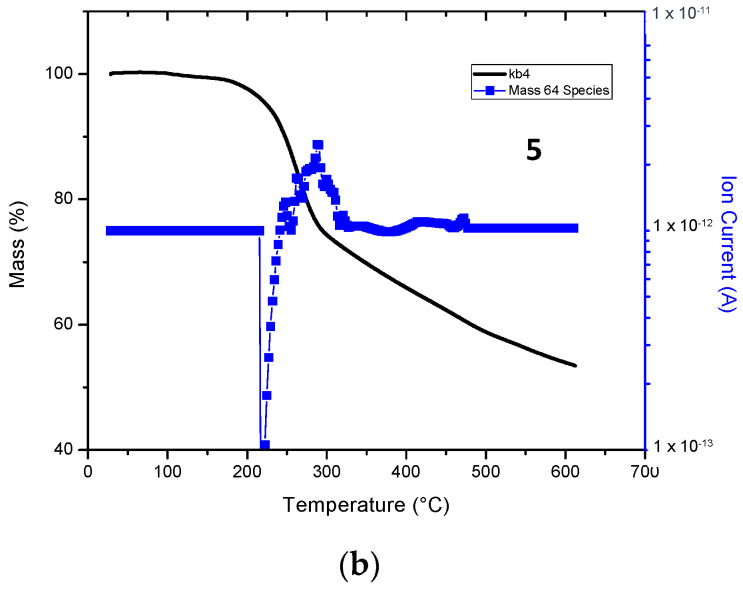
Volatile species detected by mass spectrometry (MS) during thermogravimetric analysis (TGA) of oligomers **3** and **5**. (**a**). Mass detected 19 amu with oligomer **3.** (**b**). Mass detected 64 amu with oligomer **5**.

When bulk samples of oligomers **3** and **5** were heated above 500 °C, the principal volatile product had a mass of 19 atomic mass units (amu), a mass that could be attributed to fluoride ion. On the heating of **5**, a mass of 64 amu was detected that could be derived from the loss of SO_2_ (See Figure 5).

Incomplete oxidative fluorination during preparation of intermediate **1** forms an aryl sulfur (IV) trifluoride, which is both unreactive in the addition reaction and also extremely moisture sensitive. Oliogomer chains capped with a sulfur trifluoride moiety would be susceptible to hydrolysis to the sulfinic acid on isolation of the oligomer from the reaction mixture. The loss of an ion with the mass of SO_2_ is consistent with the presence of the sulfinic acid capping group.

#### 2.5.2. Differential Scanning Calorimetry (DSC)

**Figure 6:**
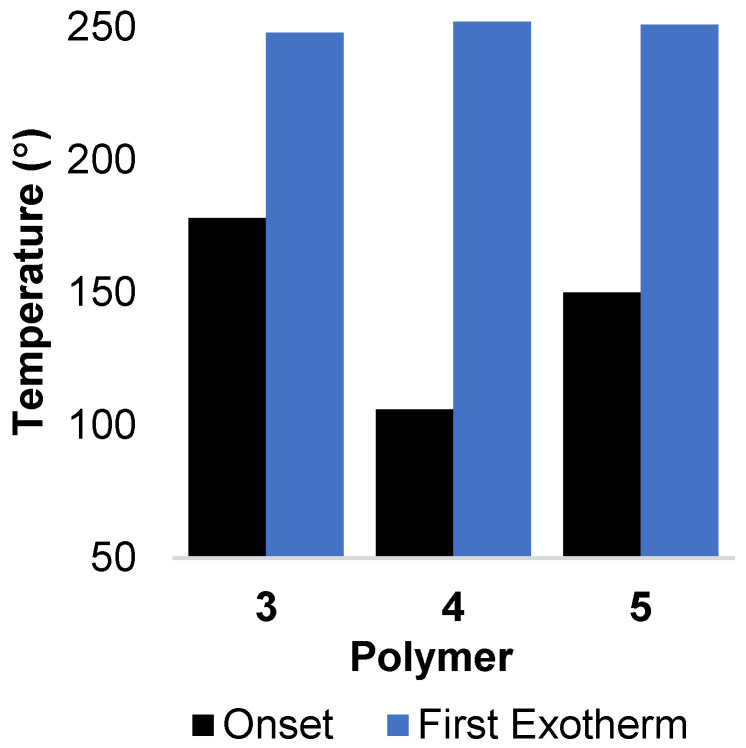
DSC data. A comparison of the temperature of the onset of the first thermal transition and the temperature of the initial exotherm maximum.

The initial exothermic events observed by DSC during heating of **3**, **4**, and **5** began as the temperature approached 100 °C, with the first pronounced exotherm occurring at 250 °C, a temperature corresponding roughly to that where the first weight loss was observed in TGA experiments (See Figure 6 and Figures S14–S16). Only very gradual mass losses were observed until final sharp weight loss and exotherm step at approximately 500–550 °C. The high thermal stability of the oligomers is consistent with the mechanical properties measured for these materials.

### 2.6. Nanoindentation Studies

Nanoindentation is an excellent method to characterize the mechanical properties of uniform thin films cast on rigid substrates [36,37]. Conventional techniques like dynamic mechanical thermal analysis or tensile testing are impractical for the evaluation of adherent thin films formed on materials such as silicon. Deformation of the thin film on a nanometer scale enables direct measurement of the mechanical properties.

Nanoindentation studies of thin films of polymers of Kevlar [23], polymethylmethacrylate PMMA [34], teflon [25], and polystyrene [24] on rigid substrates have been reported. The elastic moduli of thin polymer films and coatings are typically below 10 GPa when determined by nanonindentation. It is well-known that the moduli of polymeric films deposited on a hard substrate vary from those determined by bulk material measurements [24]. Comparison between bulk and thin film moduli can be difficult to determine with reasonable levels of uncertainty when only making shallow indents at the surface of a film [38]. For example, the strong intramolecular hydrogen bonding between fibers of Kevlar in polymer films are half of what is anticipated for the bulk material. This is a clear indication that the surface morphology can profoundly influence long range intramolecular order and hence the film modulus [23]. Elastic moduli of thin films of poly(methyl methacrylate), polystyrene, polycarbonate, and poly(vinyl chloride) on a silicon substrate have been reported to be 5.9 GPa, 5.2 GPa, 3.0 GPa, and 3.9 GPa, respectively [5]. As determination of the elastic properties for polymer films spun cast on hard materials like silicon can be problematic, comparative studies of the properties of the novel experimental samples with identically prepared, commercially available materials is especially informative.

#### 2.6.1. Nanoindentation Studies of Thin Films of **3**, **4**, and **5**

**Figure 7:**
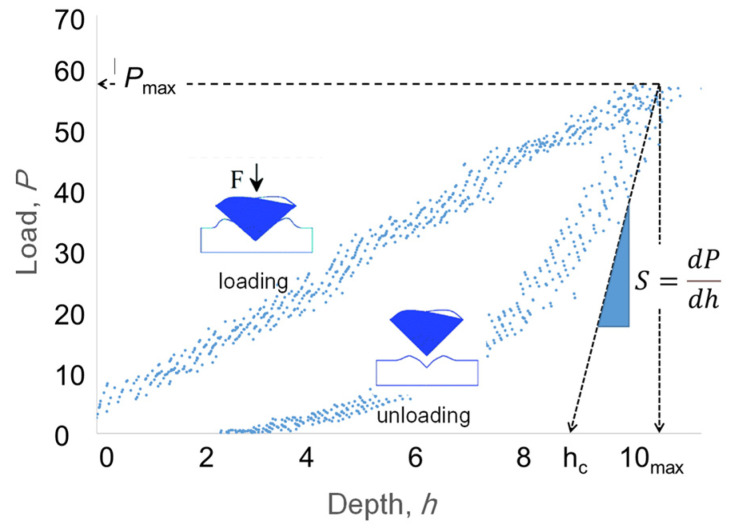
Loading and unloading profile at a depth of 10 nm for one indent measured for tetrafluorosulfanyl-containing polymer **3**.

In this work, a pyramidal Berkovich tip was used to make indents in thin films of **3**, **4**, and **5** on silicon while continuously recording the indentation load P and displacement H during one complete cycle of loading and unloading. As the indenter was driven into the film, elastic and plastic deformation of the specimen occurred. The plastic deformation resulted in the formation of a permanent hardness impression that formed to the shape of indenter at the predetermined contact depth hc (see Figure 7). As the indenter was withdrawn, elastic displacements were recovered. Analysis of the measured elastic displacement was used to predict an effective elastic modulus for the projected contact area [36,37].

Modulus is defined as the resistance to deformation as measured by the initial stress divided by the indentation depth [36].

(2) $$S=\frac{dP\;}{dh}=\frac{2}{\sqrt{\pi }}\;E_{r}\;\sqrt{A}\;$$

On rearrangement:

(3) $$E_{r}=\frac{\sqrt{\pi }}{2}\frac{S}{\sqrt{A}}$$

*S* = experimentally measured stiffness from highest slope of unloading curve

*P* = applied load

*h* = indentation depth

*E_r_* = is the reduced modulus

*A* = area of the elastic contact

Measuring the depth profile of the elastic modulus from near surface to bulk will reflect the surface state of the film [24] (See Figure 7). Elastic deformation can be recovered at depths less than 100 nm; however, exaggerated modulus values may result from the probe sensing the substrate. To ameliorate these concerns, we tested thicker films where the indentation depths were restricted to the upper 10% of the film. The values of the physical properties extracted from the resultant load–displacement curves [9] were nonetheless enhanced by the substrate. As mentioned earlier, with the immediate aim being to test thin tetrafluoro-λ^6^-sulfanyl polymer films, the properties of those films were compared directly with PMMA control films with similar thickness and also cast on a silicon substrate. With the properties of PMMA well-known, general comparisons of the mechanical properties of the films relative to those reported in the literature are readily possible.

#### 2.6.2. Polymer Moduli

**Figure 8:**
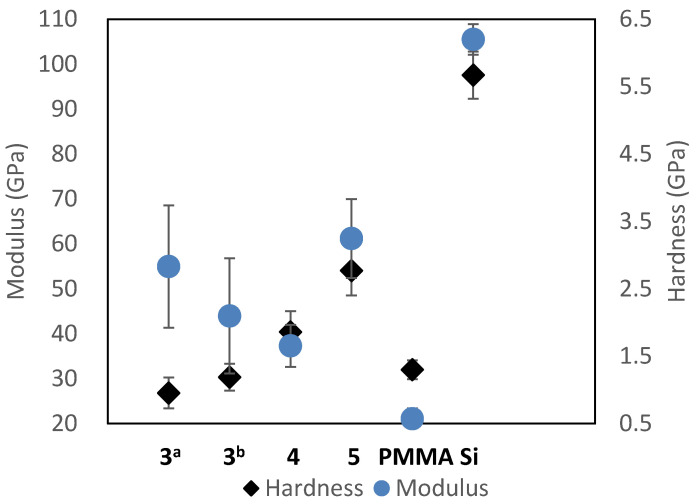
The influence of polymer structure on modulus of elasticity and hardness. Polymer **3^a^** has an M*w* of 2.3 kDa and an M*n* of 900 Da, and **3^b^** a measured M*w* of 5.1 kDa and an M*n* of 2.2 kDa. The properties of the oligomers are compared to a 314-nm-thick PMMA control film tested identically by nanoindentation. A silicon substrate was used as a control.

High thermal and chemical stability is often associated with enhanced mechanical performance [39]. The moduli of 150–200 nm films of **3**, **4**, and **5** on silicon were found to be between 40–60 GPa by nanoindentation (See Figure 8). Isomers of either monomer, as with **4** or **5**, had similar local moduli at the surface of the thin films. As previously discussed, by restricting penetrations to depths less than 10% of the film thickness, i.e., to 10 nm when characterizing 150–200 nm thick films, the elastic modulus was consistent with the bulk modulus [24]. The measured modulus values of thin films of commercially available PMMA on silicon were used as comparators. The average moduli of **3**–**5** were double to triple that of the PMMA control. Variation in dispersivity had limited influence on the stiffness of the polymer chains.

#### 2.6.3. Hardness

Hardness is the measure of resistance to localized plastic deformation induced by either mechanical indentation or abrasion. Indentation hardness measures the resistance of a sample to material deformation due to a constant compression load from a sharp object. The data utilized to acquire information on the modulus can also be used to establish the hardness, *H*, of the sample

(4) $$H=\frac{P_{max}}{A}$$

*P_max_* = max applied load

*H* = hardness

*A* = area of the elastic contact

From the experiments used to determine the modulus of films of **3**, **4**, and **5**, it was also possible to determine the resistance of the samples to elastic deformation (Figure 8). The measured hardness of 1.0–2.8 GPa was nearly double that of similar PMMA films. The meta isomer derived oligomer **5** had the highest measured hardness of 2.8 GPa. Oligomer **5** is inherently flexible and may readily align as a result of interactions at the film surface when under force from the indenter. Oligomers **4** and **5** may pack uniformly at the surface, rendering the surface topology resistant to plastic deformation, and hence be approximately 1.5 times harder than films of PMMA or **3**.

Variations in molecular weight expected for a step-growth polymerization, e.g., as with samples **3^a^** and **3^b^**, had limited influence on the stiffness of the polymer chains. Isomer substitution had similar effects on the modulus of the film. The oligomer films demonstrated modulus values above 10 GPa, suggesting strong intramolecular interactions were occurring. The glass transition temperatures observed in the thermal data suggested ordering during heating of the polymer sample. Fluidity of the polymer chains under stress and heat may be similarly independent of the isomer substitution. Commercially available PMMA was again used as a comparator.

### 2.7. UV Photocuring Influence on Modulus and Hardness

#### Radiative Curing and Orientation

**Figure 9:**
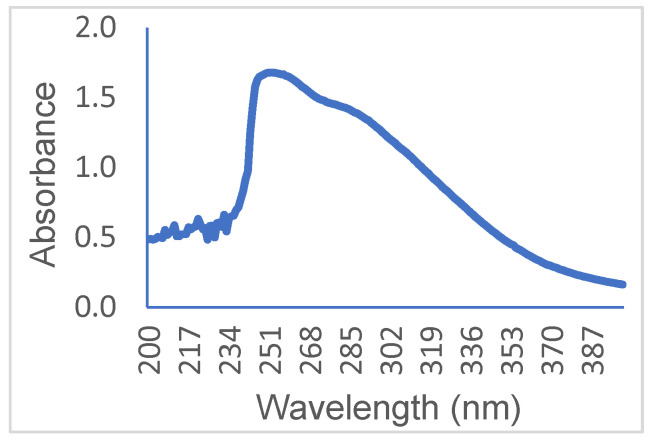
Oligomer **3** UV–visible absorbance spectra. Oligomer **3** (0.5 mg) was dissolved in 3 mL of 1,4-dioxane. The UV–visible absorbance of the oligomer was shown to be consistent with the photoreactivity of **3** on exposure to 200–450 nm radiation.

The tetrafluorosulfanyl-containing oligomers prepared as described above demonstrate a remarkable photosensitivity, a sensitivity that is likely a consequence of the π–π stacking between electron deficient and donating aromatic rings. The absorbance band measured for **3**, 250–330 nm, is consistent with the reaction promoted by exposure to UV–visible light (220–450 nm) (Figure 9).

**Figure 10:**
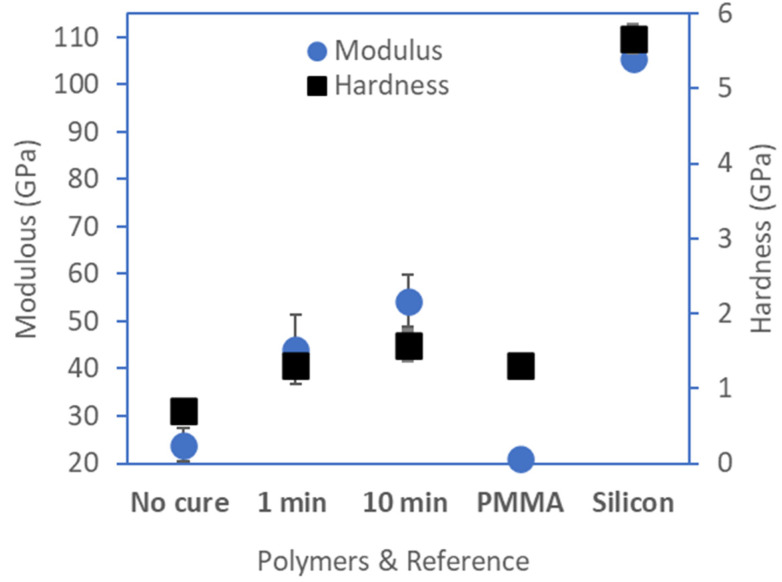
The modulus and hardness of films of oligomer **3** uncured and cured for both 1 min and 10 min are compared to the modulus and hardness of a PMMA film and the silicon substrate.

This π–π stacking interaction can be hypothesized to be the etiology of the high modulus values found. Previously, we have reported the lithographic potential of new tetrafluoro-λ^6^-sulfanyl bearing oligomers on irradiation [4]. In that report, the broad UV sensitivity of **3** was demonstrated [4]. Given that the initial photoaddition reactions of a chlorotetrafluorosulfanyl arene to an alkyne required formation of a charge transfer complex, it was anticipated that such interactions could also lead to photoinduced crosslinking to yield harder films. On exposure to 200–450 nm light, thin films of **3** are stronger and more resistant to applied mechanical loads than unexposed films (See Figure 10). Enhanced charge transfer reactions were anticipated to result in increased crosslinking and hence in hardened, cured coatings.

Both the chemical constitution of the polymer backbone and the alignment of photosensitive groups are crucial to the performance of light-cured coatings [27,40,41,42,43]. The modification of photoreactive thin films on the surface without changing the morphology of the bulk material is known [44]. Typically, photoresponsive thin films require installation of distinct and selective photochemically reactive functional groups and are commonly encountered in high performance coatings, adhesives, inks, and photoresists [15,18,19,28,29,30,31,32,33,34,45,46,47,48,49].

Following exposure to broadband UV irradiation, thin films of **3** were tested for mechanical properties.

The hardness and modulus of the film following UV curing increased with increasing UV dose relative to the uncured control **3** (See Figure 10). Moduli of our experimental samples are approximately three times more stress resistant compared to the control PMMA film. Hardness values post-radiative curing show only a modest improvement relative to the PMMA control, but there is substantial hardening compared to the uncured parent oligomer. This outcome suggests the curing step alters the surface of the oligomer film.

The glass transitions observed in the thermal data suggest ordering during heating of the polymer sample. Fluidity of the polymer chains under stress and heating may be comparable. Polymer films with modulus values greater than 10 GPa suggest strong intramolecular interactions are present. Molecular chains orient and align to assure uniform packing to minimize electronic and steric impacts.

### 2.8. Wettability

Wettability is a measure of the film surface energy, i.e., whether the polymer strands on a surface are loosely associated. Loosely associated polymer molecules can exhibit diminished wettability. Wettability is determined by water contact angle (WCA) measurements. A comparison of the WCA of films **3** and **4** to commercially available polymers can be informative. Surface packing and the morphology of the linear chains previously invoked in discussions of hardness and modulus may reduce the WCA, while the presence of the backbone tetrafluorosulfanyl group to polarize the arene subunits may also reduce the WCA.

**Figure 11:**
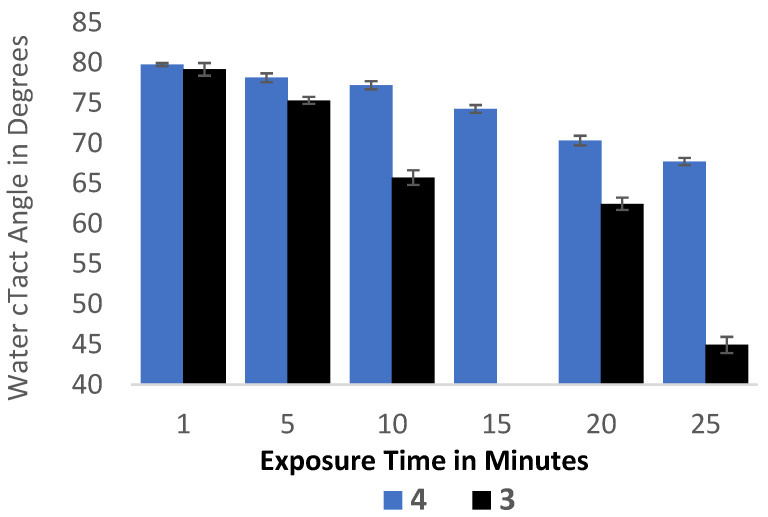
Decrease in the water contact angle of **3** and **4** after 25 min of exposure to UV irradiation.

Thin films of **3** and **4** were cast and the wettability determined. The contact angle was determined to be 75 degrees on testing with deionized water (Figure 11). The wetting behavior of the thin films suggests moderate hydrophobicity, similar to values determined for polyamides, epoxies, and polysulfones. Following UV exposure for 25 min, the hydrophobicity of the films decreased with a WCA of 45 degrees for **3** and 67 degrees for **4**.

## 3. Materials and Methods

### 3.1. Materials and Reagents

Chemicals were purchased from Sigma Aldrich and used as received unless otherwise noted. Tetrafluorosulfanyl chlorides subsituted at the meta and para positions were prepared according to the literature [1]. Acetonitrile was distilled and stored over 4 Ǻ molecular seives. Silicon wafers were purchased from University Wafer (South Boston, MA 02127, USA) for NI experiments.

### 3.2. Material Preparation

The stoichiometric quantities of a single isomer of a bis(chlorotetrafluorosulfanyl)arene was mixed with a single isomeric dialkynylarenes in dry diethyl ether to produce the oligomer. The mixture was allowed to oligomerize for 24 h using a compact fluorescent light source. The polymerization was followed using ^19^F NMR to confirm the disappearance of the starting bis(chlorotetrafluorosulfanyl)arene (δ 135 ppm) and appearance of polymer product (δ 70 ppm). The diethyl ether was evaporated under reduced pressure, resulting in a sand-colored solid. The solids were dissolved in a minimal volume of dichloromethane. Ethanol was added to precipitate a yellow-tan solid polymer from the stirring solution. Solids were isolated by filtration and were dried under reduced pressure. Oligomers were characterized by ^1^H, ^13^C, and ^19^F NMR and IR spectroscopy (See Figures S2–S13 and Tables S1–S3).

### 3.3. Infrared Spectroscopy

A PerkinElmer UATR Two FT-IR Spectrometer and PerkinElmer Spectrum 100 FT-IR Spectrometer (PerkinElmer, New York, NY, USA) were used for sample analysis Figure S12. Solid polymer (1–2 mg) was tested following precipitation. FTIR ν = 3296 (w), 1602 (w), 1501 (w), 1402 (w), 1214 (w), 1148 (w), 1091 (w), 1013 (w) 913 (s), 882 (s), 836 (m), 751 (s), 665.95 (s), 628 (m), 574 (w), 482 (w), 406 (w) cm^−1^ (See Figures S11–S13 and Tables S1–S3).

### 3.4. Thermal Characterization

The polymeric material (5–7 mg) was tested for thermal stability using a TA Instruments TGA 2050 Thermogravimetric Analyzer, New Castle, DE USA with a platinum pan and a heating rate of 10 °C/min. Differential scanning calorimetry (DSC) was determined using a TA Instruments DSC 2920 Differential Scanning Calorimeter, New Castle, DE USA and a heating rate of 10 °C/min (See Figures S14–S16).

### 3.5. Gel Permeation Chromatography (GPC)

The polymer was precipitated using ethanol or methanol and dried under reduced pressure. The solids were dissolved in HPLC-grade THF in a ratio of 1–4 mg per mL of solvent. Samples were passed through a 25 mm Acrodisc^®^ syringe filter before analysis. Molecular weight measurements were performed using a Viscotek GPC equipped with three Viscotek T6000M columns and a Viscotek model 302 tetra detector array, Malvern, United Kingdom, at 35 °C and a flow rate of 0.8 mL/min with THF as the mobile phase. The instrument was calibrated using polystyrene standards (See Figures S17–S19).

### 3.6. Sample Preparation for Nanoindentation Studies

Thin films were prepared using the drop cast method. Solids were solubilized in 1,4-dioxane to a 2–4 wt.% concentration and were then filtered using a 0.45 μm Teflon filter. Thin films were cast onto piranha cleaned silicon wafers and measured by ellipsometry to be 150–200 nm in thickness. Thicknesses were measured using spectroscopic ellipsometry. Thickness measurements were acquired using a J. A. Woollam M-2000 fixed-angle ellipsometer, Lincoln, Nebraska equipped with Complete Ease 6 software, Lincoln, Nebraska The thicknesses were fitted using a Cauchy model. Wafers were cut to approximately 5 × 5 mm squares and taped to the nanoindentation sample holder.

### 3.7. Nanoindentation (NI) Measurements

Nanoindentation experiments were performed with a Hysitron TriboIndenter (Hysitron Inc., Minneapolis, MN, USA) in a load-controlled instrument with a load resolution of 100 nN and displacement resolution of 1 nm. For measurements, a 100 nm Berkovich tip (TI-0039, Surface & Surface systems+technology GmbH, Hueckelhoven Germany) was used. The contact area function parameters were obtained from a series of nanoindentation tests performed on a fused silica standard (S/N 5-0098, Hysitron Inc., Minneapolis, MN, USA. as instructed by the instrument manufacturer. Quasi-static nanoindentation testing was performed with a displacement control for 10 nm in 10 s loading and 10 s unloading (20 s total) time profile. Each sample was tested with 16 indents (4 × 4 grid with 5 µm separation). The reduced modulus (E_r_) and hardness (H) of the indents were calculated and averaged over 16 indents per sample. The displacement was held constant at 10 nm to avoid substrate effects. Surface homogeneity is very important at 10 nm displacement. Different positions were tested on the same wafer and gave consistent results. The influence of a hard solid support is well-documented and, in an attempt to address this, we opted for shallow indents of 10 nm. This kept the indents in the upper 10% of the consistent polymer film.

## 4. Conclusions

Novel polymer architectures were characterized thermochemically and mechanically, and for wettability as well. The steric and electronic features of these new oligomers containing the -SF_4_- group would induce the surface alignment of oligomers that were spun cast as 200 nm films. Surface confinement and steric barriers to rotation create robust spun cast materials on silicon with hardnesses of 1.5 GPa and a moduli of 40–60 GPa. Upon curing with UV irradation, the films were rendered 2 times harder and stiffer. Longer curing times resulted in less hydrophobic films that may nonetheless have utility in advanced patterning, automotive, display, and textile applications.

## Figures and Tables

**Table 1:** Gel permeation chromatography results.

Polymer	*M*_w_ (Daltons) ^a^	*M*_n_ (Daltons)	*M*_p_ (Daltons)	Ð
3	5800	3700	3900	1.6
4	4300	1800	3700	2.4
5	3400	1800	2400	1.9

^a^ Estimated utilizing a polystyrene standard.

## Data Availability

The original contributions presented in the study are included in the article/Supplementary Material. Further inquiries can be directed to the corresponding author/s.

## Supplementary Materials

The following supporting information can be downloaded at: https://www.mdpi.com/article/10.3390/molecules29184413/s1, Figure S1: TGA oligomers **3**, **4**, and **5**; Figures S2–S10: ^1^H, ^13^C and ^19^F NMR spectra; Figures S11–S13: IR spectra; Figures S14–S16: Thermal Characterization Data; Figures S17–S19: Gel Permeation Chromatography; Figure S20: Water contact angles for **3**, **4** and **5**; and Table S1–S3: IR stretches.
