# Supplementary material for: Photocurable Hypervalent Fluorinated Sulfur Containing Thin Films with Remarkable Hardness and Modulus
Source: Molecules. 2024 Sep 17;29(18):4413. doi: 10.3390/molecules29184413 (PMC11434361; doi:10.3390/molecules29184413)
Supplement: Supplementary file 1 [file molecules-29-04413-s001.zip › molecules-3138765-supplementary.pdf]

((Supporting Information can be included here using this template))

Supporting Information

Photocurable Hypervalent Fluorinated Sulfur Containing Thin Films with Remarkable Hardness and Modulus

*Kelly A. Bonetti[1], Denis Rende[3],Michael Murphy[2], Prof. John T. Welch[1,]**

Contents

Thermogravimetric Analyses 3
Figure S1. TGA polymers **3, 4** and **5** **3**
NMR Characterization 4
Figure S2. ^{1}H NMR **3** 4
Figure S3. ^{19}F NMR **3** 4
Figure S4. ^{13}C NMR **3** 5
Figure S5. ^{1}H NMR **4** 5
Figure S6. ^{19}F NMR **4** 6
Figure S7. ^{13}C NMR **4** 6
Figure S8. ^{1}H NMR **5** 7
Figure S9. ^{19}F NMR **5** 7
Figure S10. ^{13}C NMR **5** 8

Infrared Characterization 9
Figure S11. IR Analysis **3** 9
Table S1. IR stretches **3** 9
Figure S12. IR Analysis **4** 10
Table S2. IR stretches **4** 10
Figure S13. IR Analysis **5** 11
Table S3. IR stretches **5** 11

Thermal Characterization 12
Figure S14. TGA and DSC data **3**. 12
Figure S15. TGA and DSC data **4**. 12
Figure S16. TGA and DSC data **5**. 13

Characterization by Gel Permeation Chromatography 14
Figure S17. GPC Analysis **3**. 14
Figure S18. GPC Analysis **4**. 15
Figure S19. GPC Analysis **5**. 16

Water Contact Angle Measurements 17
Figure S20. Water contact angles for **3, 4 and 5.** 17

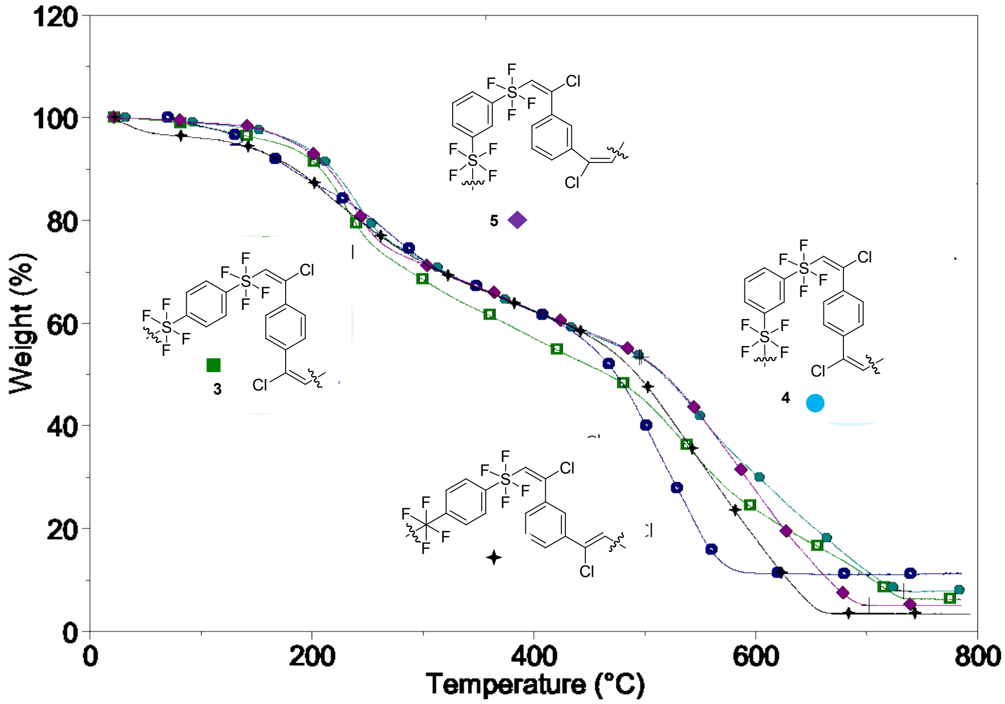

Figure S1. Thermogravimetric analyses of polymers **3, 4** and **5. ** Tetrafluorosulfanyl-containing polymers **3**, **4** and **5** demonstrate two major weight losses..

NMR Characterization

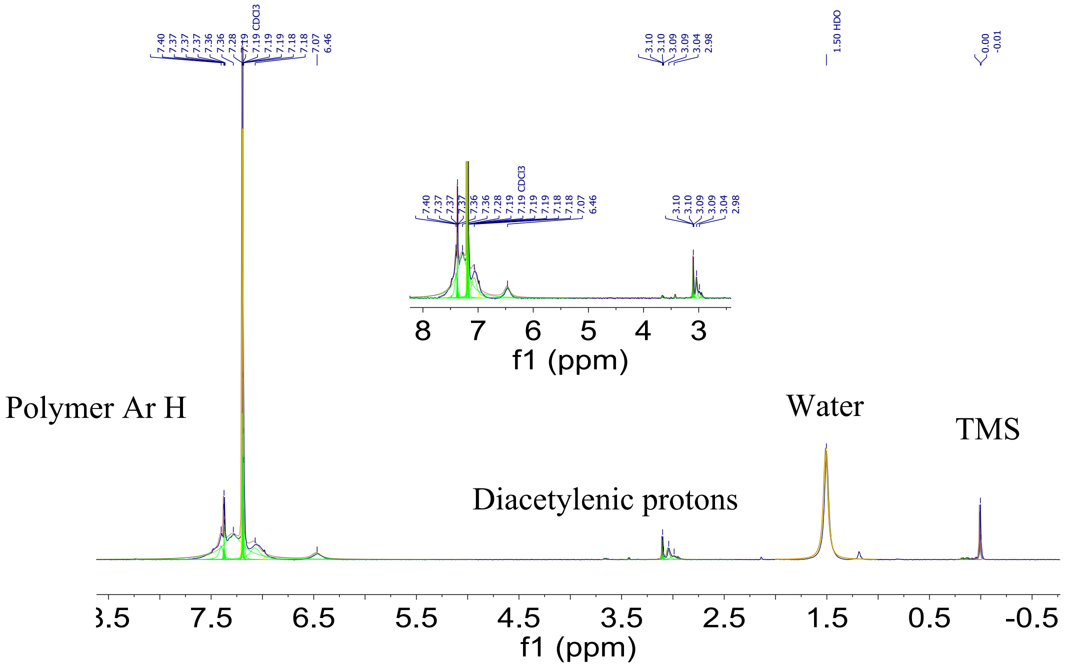

Figure S2. ^{1}H NMR polymer **3** in chloroform-*d*.[1]

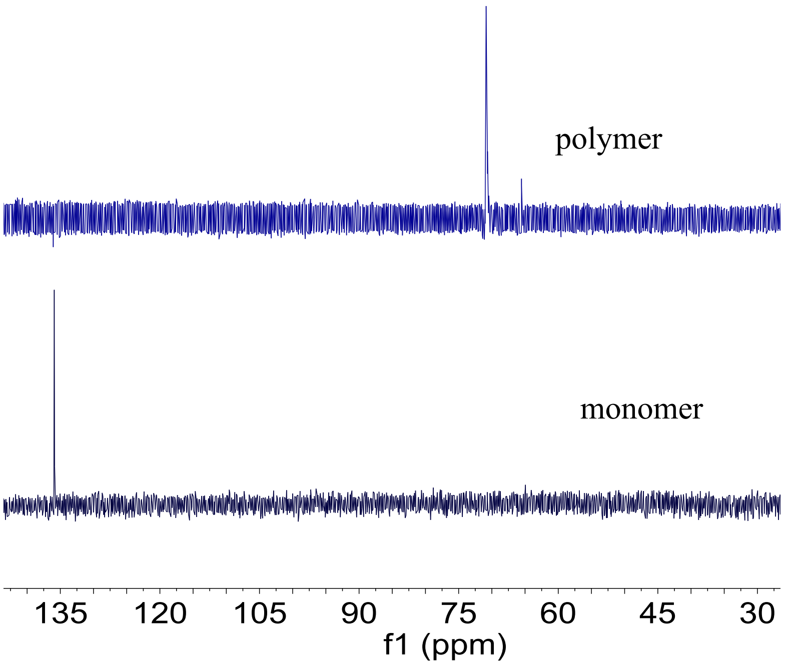

Figure S3. ^{19}F NMR of monomer **1** compared with polymer **3** with baseline smoothing. Samples dissolved in chloroform-*d*.[1]

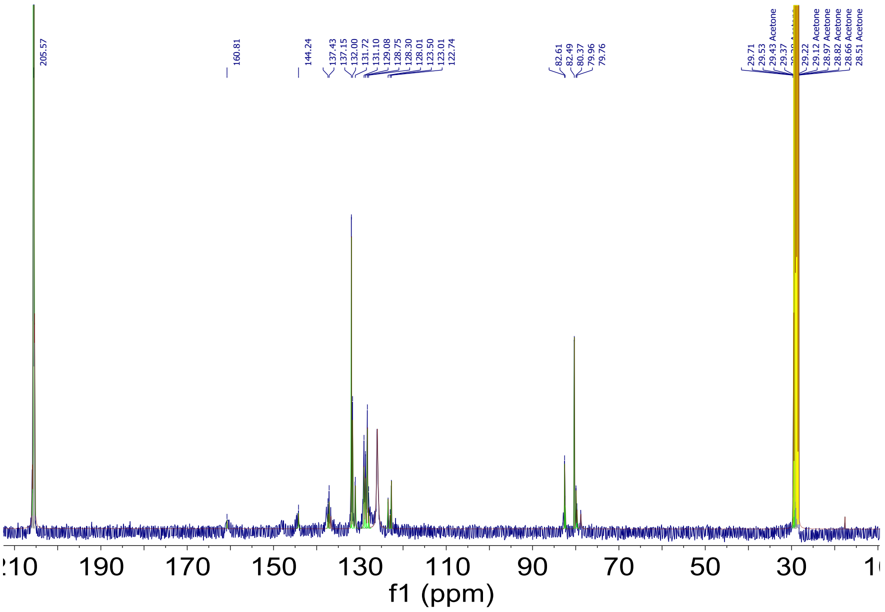

Figure S4. Polymer **3** ^{13}C in acetone-d^6. ^{13}C (500 MHz, acetone-d^6): δ 160.8 (p, 2C, $ArSF_4Cl$), 144.2 (m, 2C, alkene SF_4-C), 137.2 (m, 2C, alkene SF_4-C), 132.0 (s, 4C, Ar C), 131.7 (s, 4C, Ar C), 131.09 (s, 4C, Ar C), 129.1 (p, 2C, alkene C-Cl), 128.7 (m, 2C, Ar C), 128.3 (m, 2C, Ar C), 126.1 (m, 2C, Ar SF_4), 123.5 (s, 2C, Ar C), 82.6 (s, 1C, alkyne C), 79.8 (s, 1C, Alkyne C).[1]

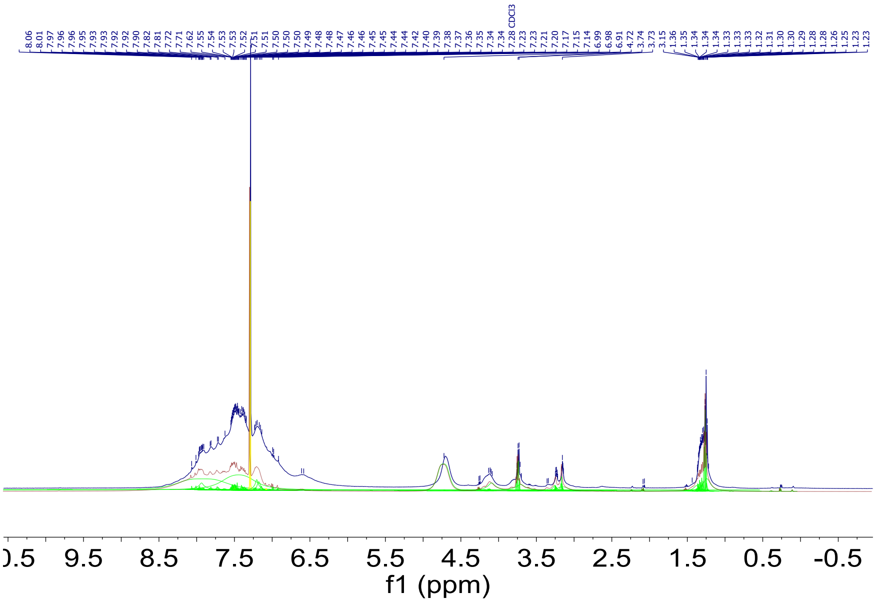

Figure S5. ^1H NMR of polymer **4** in chloroform-*d*.

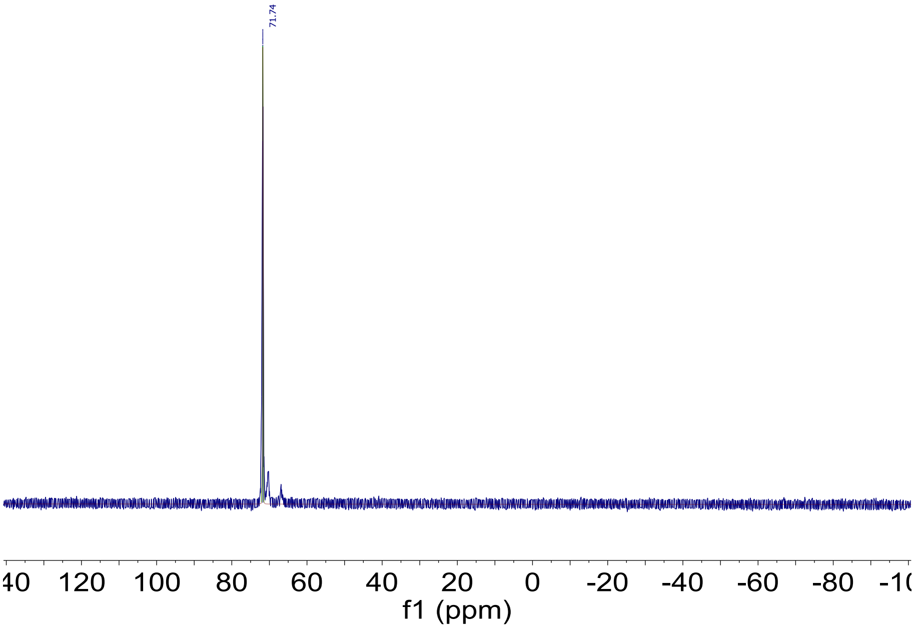

Figure S6. ^{19}F NMR of polymer **4** in chloroform-*d*.

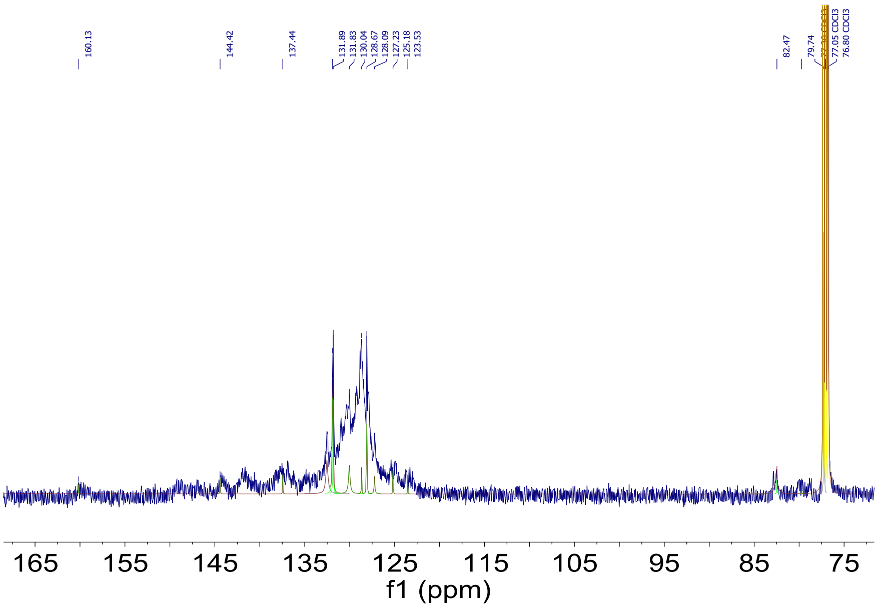

Figure S7. ^{13}C NMR of polymer **4** in chloroform-*d*.

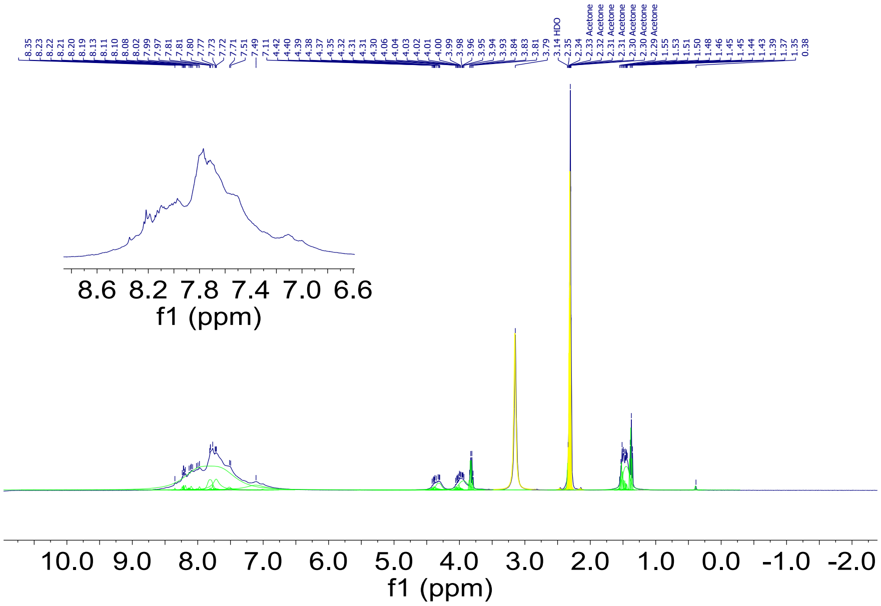

Figure S8. ^{1}H NMR of polymer **5** in acetone-d^6.

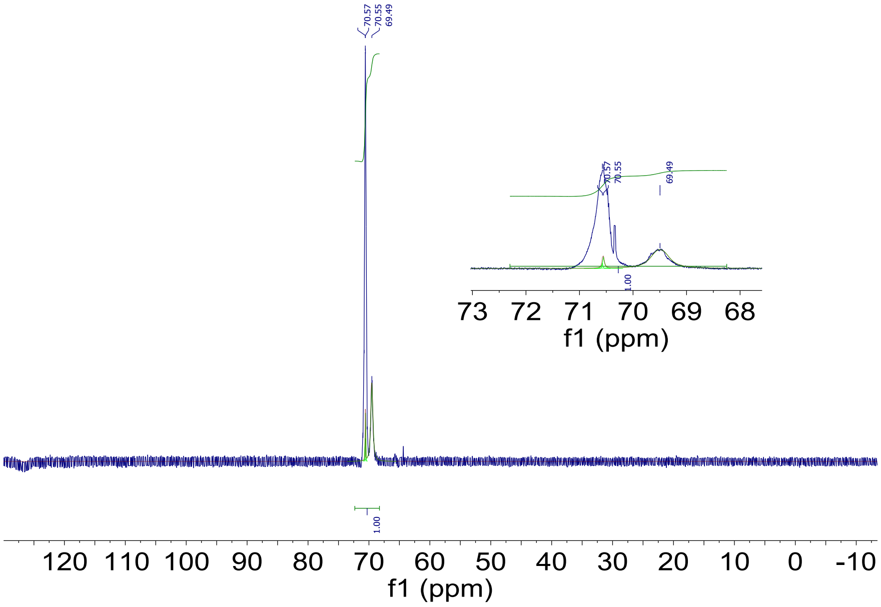

Figure S9. ^{19}F NMR of polymer **5** in chloroform-d

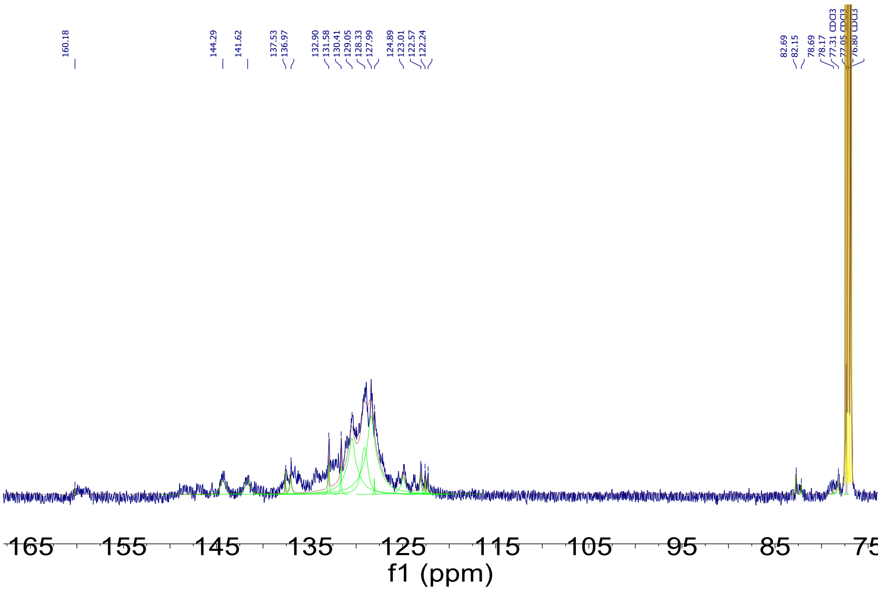

Figure S10. ^{13}C NMR of polymer **5** in chloroform-*d*.

Infrared Spectra

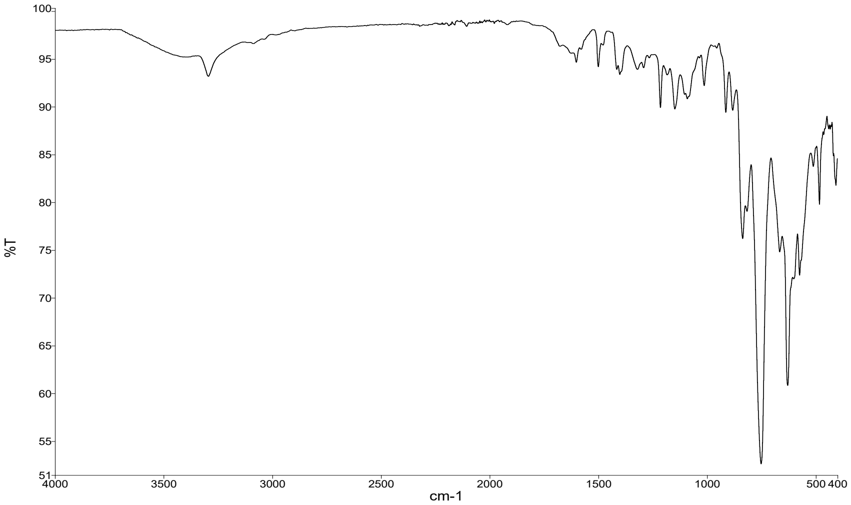

Figure S11. IR of the polymer **3**.

Table S1. IR peaks of polymer **3**.

Peak Name	X (cm-1)	Y (%T)
1	3296.21	93.25
2	1602.31	94.73
3	1501.03	94.26
4	1402.11	93.44
5	1214.9	89.98
6	1148.64	89.82
7	1091.25	90.89
8	1013.34	92.27
9	913.86	89.48
10	882.11	89.7
11	836.16	76.24
12	751.25	52.62
13	665.95	74.84
14	628.8	60.84
15	574.1	72.4
16	482.57	79.83
17	406.84	81.83

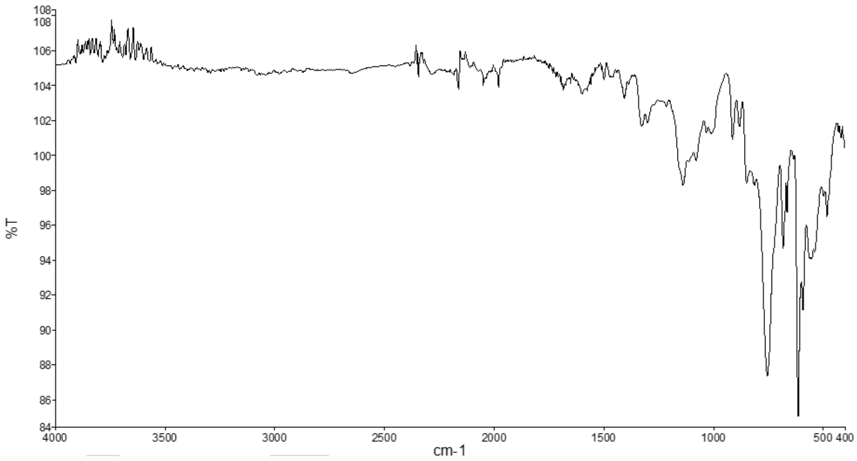

Figure S12. IR of polymer **4**

Table S2. Peak Labels of polymer **4**

Peak Name	X (cm-1)	Y (%T)
1	912.41	100.94
2	753.28	87.35
3	681.77	94.67
4	612.92	85.01
5	552.43	94.06

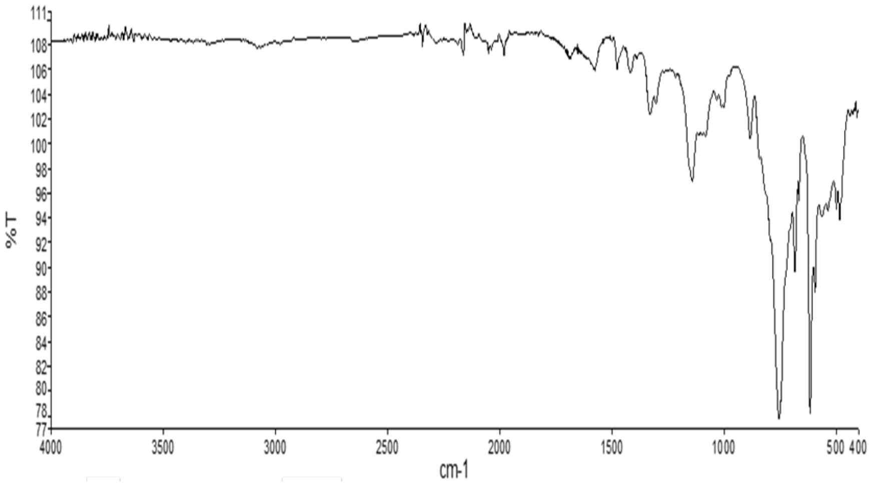

Figure S13. IR of polymer **5**.

Table S3. IR Peaks of polymer **5.**

Peak Name	X (cm-1)	Y (%T)
1	3678.64	108.44
2	3079.38	107.81
3	2345.23	107.95
4	2161.49	107.25
5	2049.75	107.41
6	1979.42	107.17
7	1682.27	106.9
8	1574.24	105.98
9	1475.17	106.08
10	1416.7	105.8
11	1328.46	102.42
12	1141.06	97.01
13	999.96	103.03
14	881.59	100.44
15	752.48	77.73
16	682.02	89.63
17	664.36	95.44
18	613.76	78.19
19	592.22	88.07
20	496.77	94.79
21	481.88	93.85

Thermal Characterization

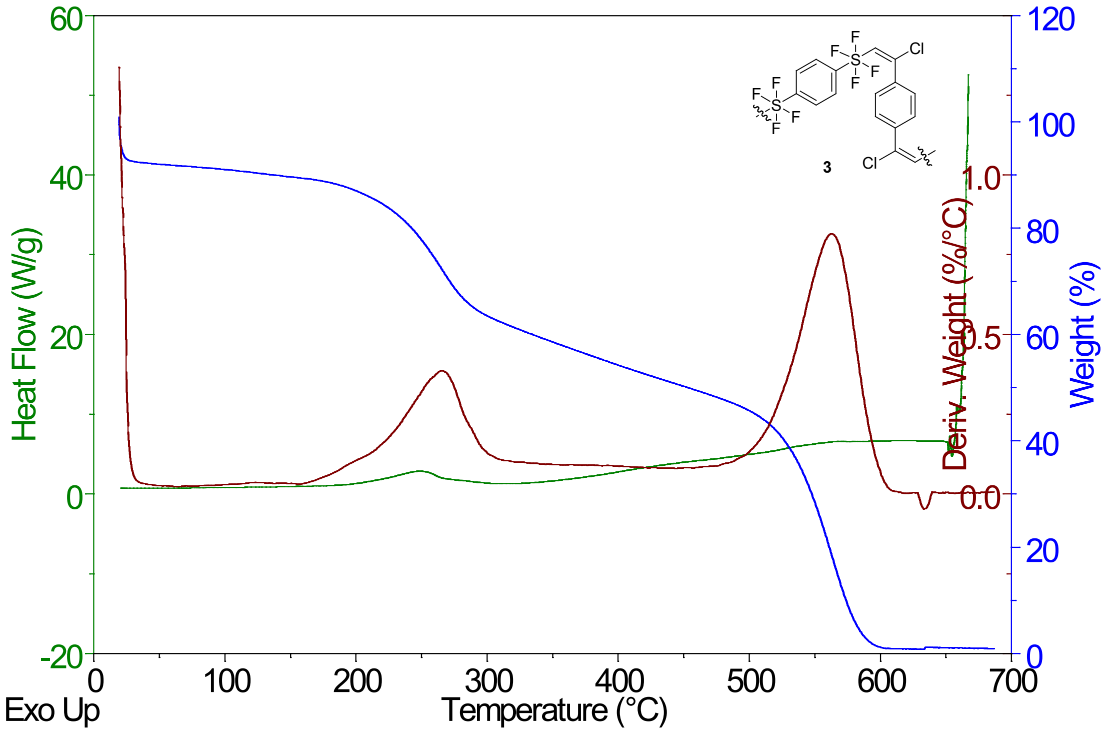

Figure S14. TGA and DSC of polymer **3**.

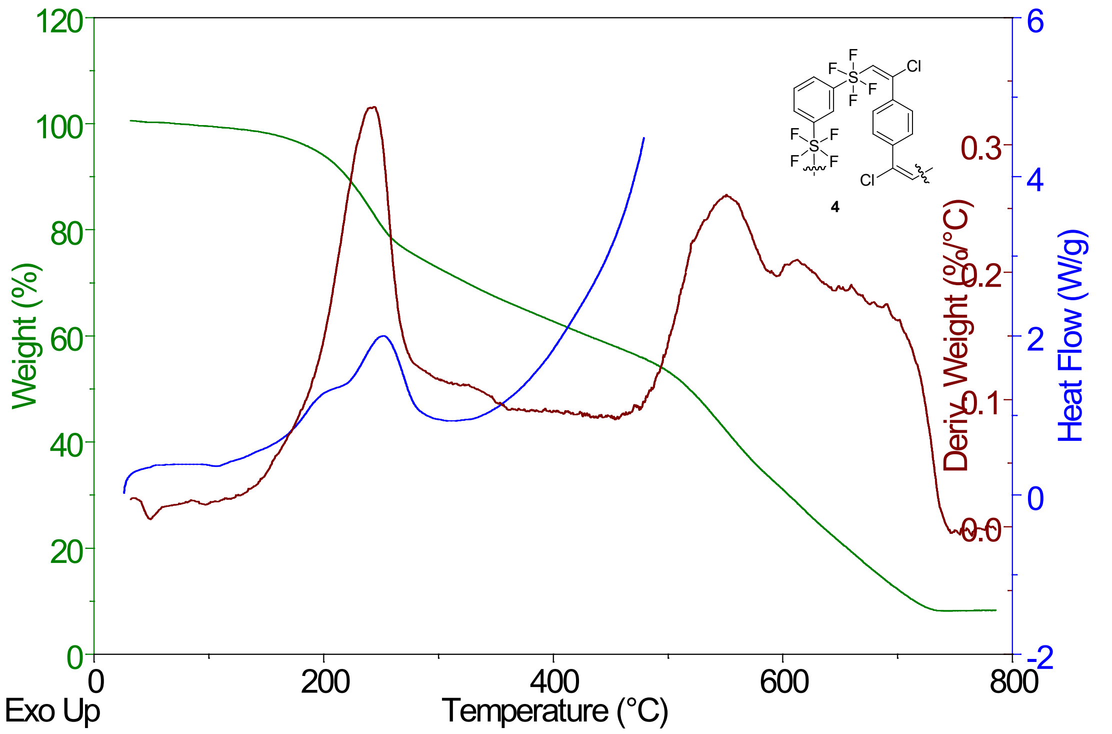

Figure S15. TGA and DSC of polymer **4**.

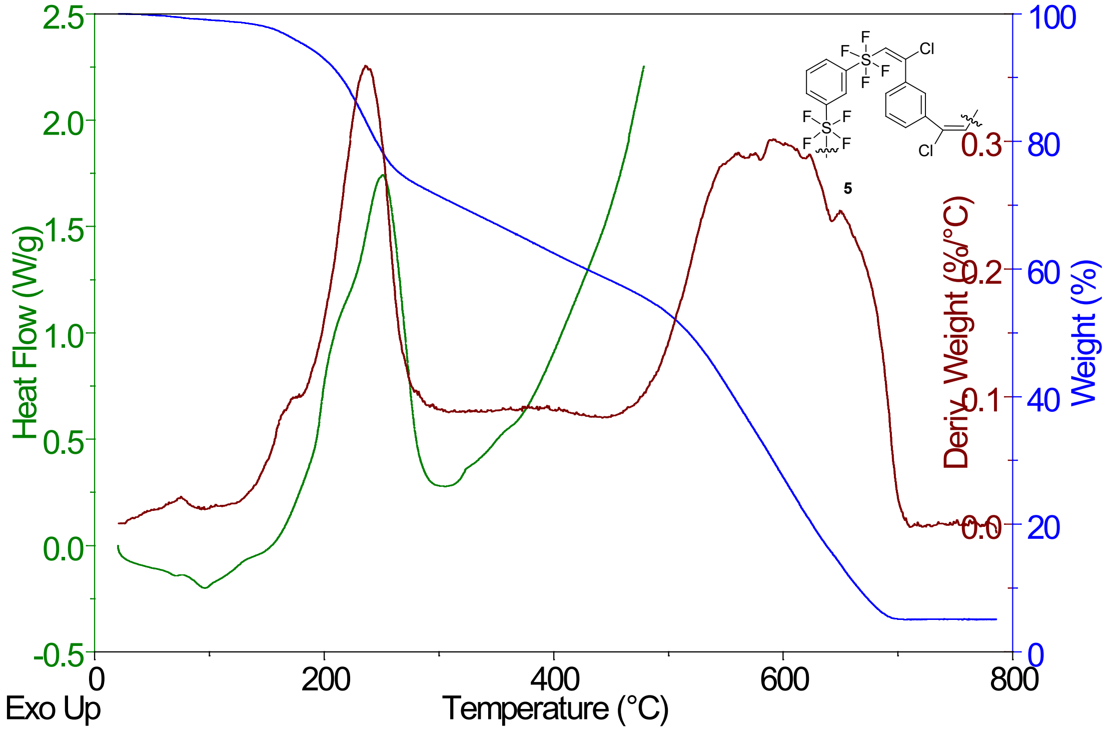

Figure S16. TGA and DSC of polymer **5**.

Gel Permeation Chromatography

Figure S17. GPC data for **3**

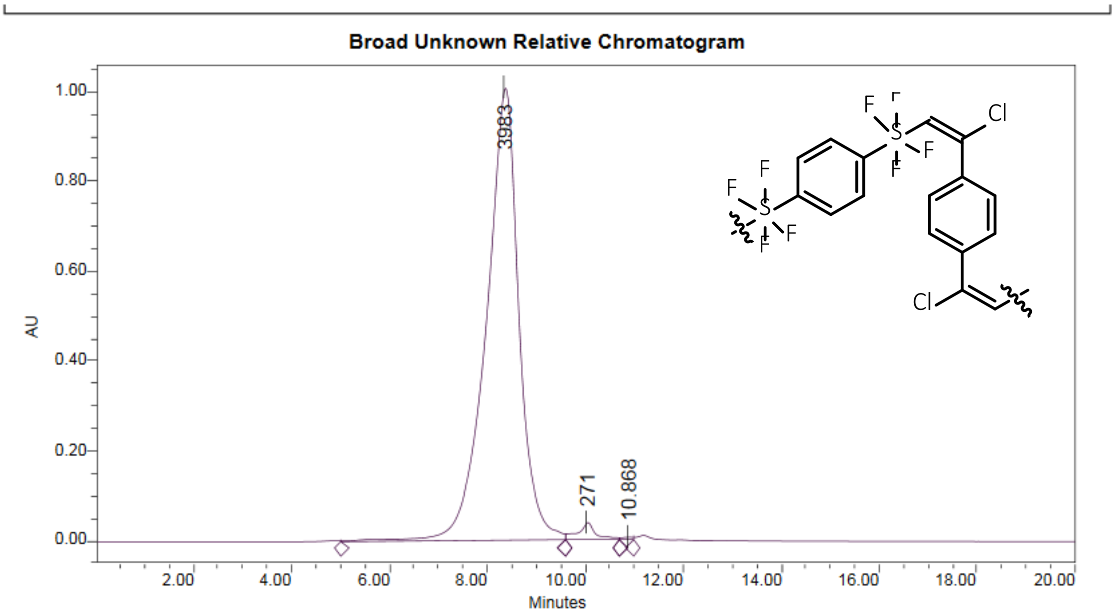

Broad Unknown Relative Peak Table

	Distribution Name	Mn (Daltons)	Mw (Daltons)	MP (Daltons)	Mz (Daltons)	Mz+1 (Daltons)	Polydispersity	Mz/Mw	Mz+1/Mw
1		3758	5833	3983	10720	22683	1.552072	1.837888	3.889080
2				271					

Figure S18. GPC data for **4**.

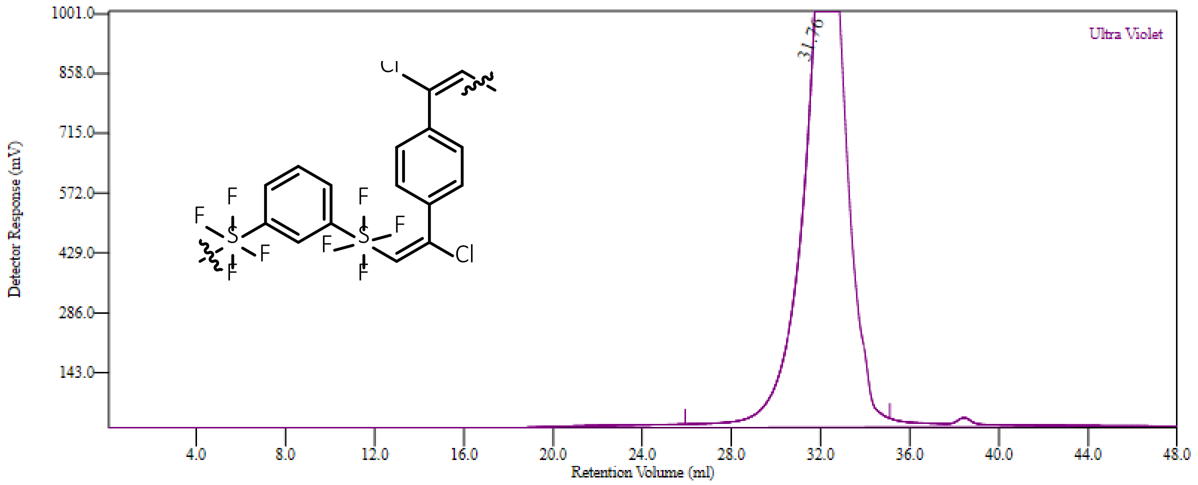

Warning: No flow rate marker was found.

Conventional Calibration - Homopolymers : Results

Peak RV - (ml)		31.760
Mn - (Daltons)		1,801
Mw - (Daltons)		4,310
Mz - (Daltons)		18,926
Mp - (Daltons)		3,716
Mw / Mn		2.393
Percent Above Mw:	0	0.000
Percent Below Mw:	0	0.000
Mw 10.0% Low		617
Mw 10.0% High		18,542
RI Area - (mvml)		0.00
UV Area - (mvml)		2541.11

Annotation	
Method File	cc-0002.vcm
Limits File	49:54_13_MONOMER_14_DEB_01-cc-0002-0000.lim
Date Acquired	Nov 16, 2018 - 15:49:54
Solvent	THF
Acquisition Operator	admin : Administrator
Calculation Operator	admin : Administrator
Column Set	GMHxl
System	System 1
Flow Rate - (ml/min)	0.800
Inj Volume - (ul)	100.0
Volume Increment - (ml)	0.00267
Detector Temp. - (deg C)	30.0
Column Temp. - (deg C)	30.0
OmniSEC Build Number	210

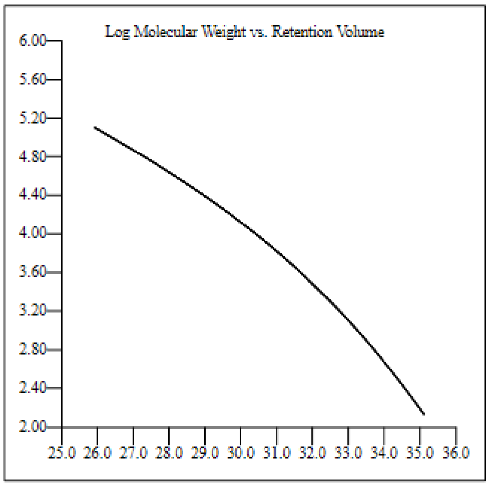

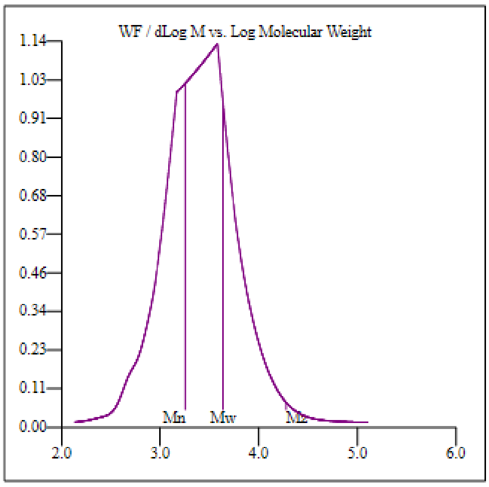

Figure S19. GPC data for **5**.

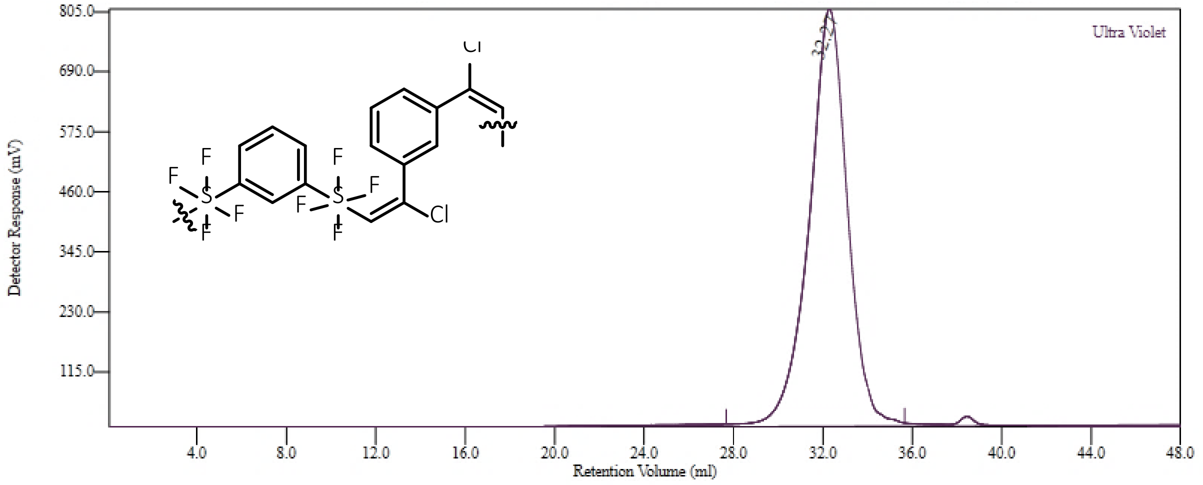

Warning: No flow rate marker was found.

Conventional Calibration - Homopolymers : Results

Peak RV - (ml)		32.275
Mn - (Daltons)		1,753
Mw - (Daltons)		3,402
Mz - (Daltons)		7,085
Mp - (Daltons)		2,429
Mw / Mn		1.940
Percent Above Mw:	0	0.000
Percent Below Mw:	0	0.000
Mw 10.0% Low		652
Mw 10.0% High		10,891
RI Area - (mvml)		0.00
UV Area - (mvml)		1587.45

Annotation	
Method File	cc-0002.vcm
Limits File	48:58_13_MONOMER_13_DEB_01-cc-0002-0001.lim
Date Acquired	Nov 16, 2018 - 14:48:58
Solvent	THF
Acquisition Operator	admin : Administrator
Calculation Operator	admin : Administrator
Column Set	GMHxl
System	System 1
Flow Rate - (ml/min)	0.800
Inj Volume - (ul)	100.0
Volume Increment - (ml)	0.00267
Detector Temp. - (deg C)	30.0
Column Temp. - (deg C)	30.0
OmniSEC Build Number	210

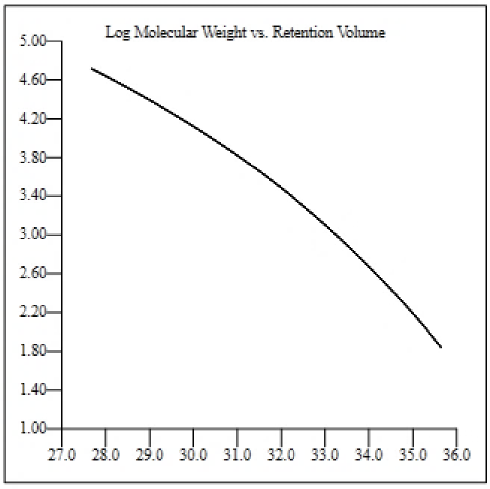

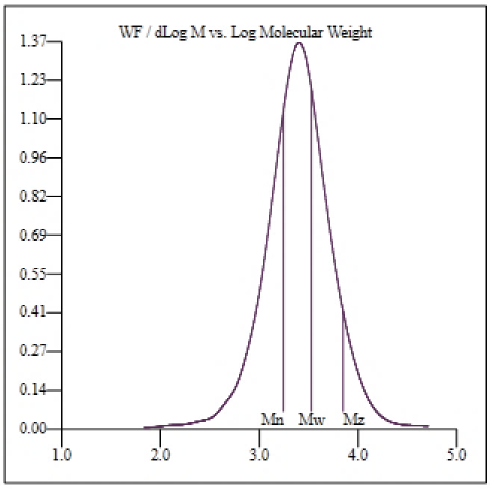

Figure S20. Water contact angles for **3,4 and 5.**.

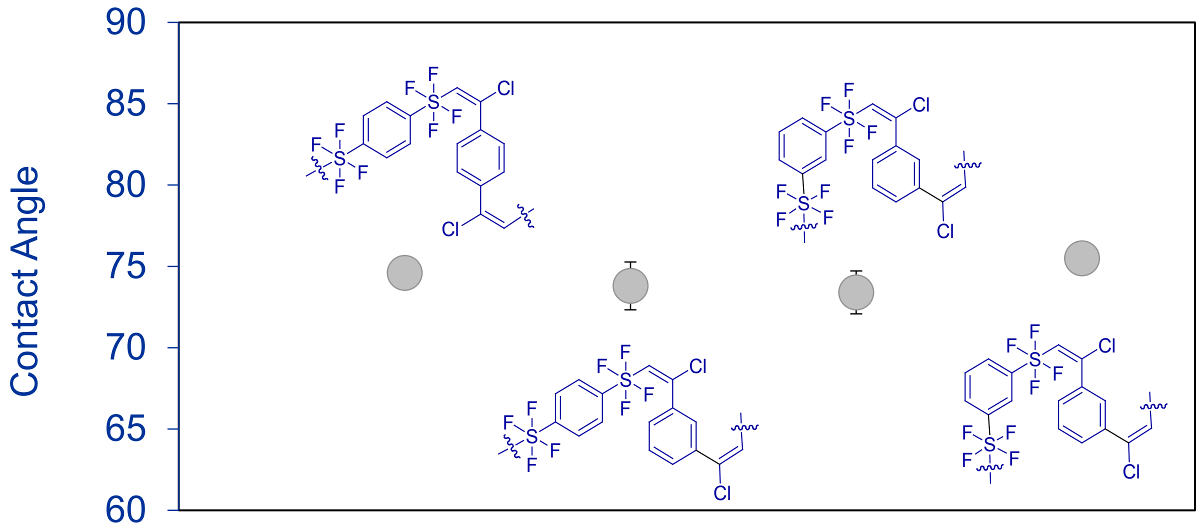

	Contact Angle
**1414**	74.6±0.49 °
**1413**	73.8±1.5 °
**1313**	73.4±1.3 °
**1314**	75.5±1.0 °

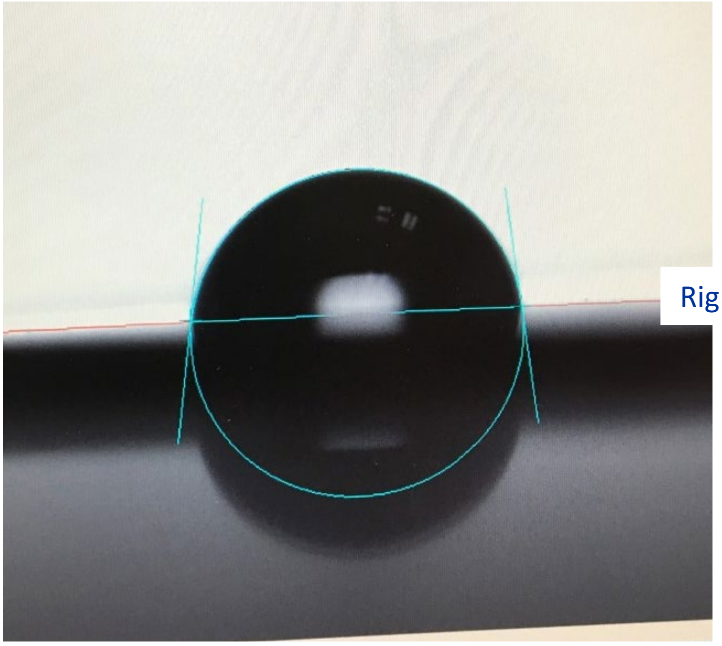

Right and left contact angle of the same water spot.

References

1. Bonetti, K.A.; Murphy, M.; Brainard, R.L.; Zhong, L.; Welch, J.T. Photosensitive Hypervalent Fluorinated Sulfur Containing Polymers for Light Sensitive Applications. *J. Polym. Sci. (Hoboken, NJ, U. S.)* **2020**, *58*, 787-791, doi:10.1002/pol.20190104.
